# Glucose deprivation regulates the progranulin–sortilin axis in PC12 cells

**DOI:** 10.1002/2211-5463.12164

**Published:** 2016-12-22

**Authors:** Ken‐ichi Kawashima, Yuri Ishiuchi, Miki Konnai, Saori Komatsu, Hitoshi Sato, Hideo Kawaguchi, Nobumitsu Miyanishi, Jérôme Lamartine, Masugi Nishihara, Taku Nedachi

**Affiliations:** ^1^ Graduate School of Life SciencesToyo UniversityOura‐gunGunmaJapan; ^2^ Department of Applied BiosciencesFaculty of Life SciencesToyo UniversityOura‐gunGunmaJapan; ^3^ Graduate School of Food and Nutritional SciencesToyo UniversityOura‐gunGunmaJapan; ^4^ LBTI UMR CNRS 5305 ‐ University Claude Bernard Lyon ILyonFrance; ^5^ Graduate School of Agricultural and Life SciencesThe University of TokyoJapan

**Keywords:** glucose, PC12 cells, progranulin, sortilin

## Abstract

Progranulin (PGRN) is a growth factor implicated in several neurodegenerative diseases, such as frontotemporal lobar degeneration. Despite its important role in the central nervous system (CNS), the mechanisms controlling PGRN expression in the CNS are largely unknown. Recent evidence, however, suggested that several stressors, such as hypoxia, acidosis, or oxidative stress, induce PGRN expression. The present study was mainly aimed at determining whether and, if so, how glucose deprivation affects PGRN expression in PC12 cells. Initially, it was found that glucose deprivation gradually induced *PGRN* gene expression in PC12 cells. To elucidate the underlying molecular mechanisms, several intracellular signalings that were modified in response to glucose deprivation were examined. Both adenosine monophosphate kinase (AMPK) activation and changes in osmotic pressure, which are modified by extracellular glucose concentration, had no effect on *PGRN* gene expression; on the other hand, p38 activation in response to glucose deprivation played an important role in inducing *PGRN* gene expression. It was also found that expression of sortilin, a PGRN receptor implicated in PGRN endocytosis, was dramatically reduced by glucose deprivation. In contrast to glucose‐dependent regulation of *PGRN* gene expression, AMPK activation played a central role in reducing sortilin expression. Overall, the present study suggests that the PGRN–sortilin axis is modulated by glucose deprivation via two distinct mechanisms. As PGRN is neuroprotective, this system may represent a new neuroprotective mechanism activated by glucose deprivation in the CNS.

**Abbreviations**
ACC: acetyl‐CoA carboxylase
AICAR: 5‐amino‐imidazole‐4‐carboxamide‐1‐β‐D‐ribofuranoside
ALS: amyotrophic lateral sclerosis
AMPK: adenosine monophosphate kinase
ATF: activating transcription factor
CNS: central nervous system
CREB: cyclic AMP‐responsive element‐binding protein
DMEM: Dulbecco's modified Eagle's medium
FBS: fetal bovine serum
FTLD: frontotemporal lobar degeneration
GEP: granulin epithelin precursor
GF: glucose‐free
HG: high glucose
HSP27: heat‐shock protein 27
LDH: lactose dehydrogenase
LG: low glucose
N2A: neuro‐2 A neuroblastoma
NGF: nerve growth factor
p75NTR: p75 neurotrophin receptor
PCDGF: PC‐cell‐derived growth factor
PGRN: progranulin
PVDF: polyvinylidene difluoride
ROCK: Rho‐associated protein kinase
SIRT1: sirtuin 1
TBS: Tris‐buffered saline
VPS: vacuolar protein sorting

Progranulin (PGRN), also known as granulin epithelin precursor, PC‐cell‐derived growth factor (PCDGF), proepithelin or acrogranin, is a growth factor that consists of 593 amino acids, expressed in various tissues including brain. It has been demonstrated that decreased PGRN levels in the central nervous system (CNS) are associated with certain types of neurodegenerative disease. For example, numerous mutations in *PGRN* gene, leading to PGRN haploinsufficiency, are occasionally observed in frontotemporal lobar degeneration and amyotrophic lateral sclerosis 1, 2, 3. Several studies clearly demonstrated that PGRN has a neuroprotective action 4, 5, 6. Moreover, although PGRN haploinsufficiency in *Grn* ^+/−^ mice displays limited neuroinflammatory phenotypes 7, evidence from *Grn* ^−/−^ mice suggested that PGRN gene deficiency fosters inflammatory changes in the CNS 8, 9.

Despite the importance of PGRN function in the CNS, the molecular basis of its action remains elusive. Several receptors for PGRN were recently proposed, including sortilin, which is a multifunctional protein belonging to the vacuolar protein sorting 10p domain receptor family 10. Because sortilin endocytoses and delivers PGRN to lysosomes, sortilin seems to contribute to reducing extracellular PGRN levels 10. In addition, several studies showed that some effects of PGRN, such as inducing neuronal outgrowth and neurotrophic properties, are independent of sortilin, suggesting that another mechanism or mechanisms may also be involved in PGRN action 11, 12. PGRN expression control is also not completely understood. Hypoxia, acidosis, and oxidative stress stimulate PGRN expression in different cell types 13, 14, 15, and PGRN can be considered as a stress‐responsive factor; however, whether other forms of stress also stimulate PGRN expression is not known.

The CNS has one of the highest metabolic rates in the body. A shortage of glucose, as often observed, for example, during ischemia, decreases neuron viability and eventually causes cell death, by either necrosis or apoptosis 16, 17, 18, 19. Recent evidence, however, demonstrated that glucose deprivation not only leads to cell death but also exerts several protective mechanisms to counteract these adverse effects. For instance, transient glucose deprivation was reported to confer a preconditioning‐like protection against subsequent detrimental stress such as ischemic reperfusion 20. We also recently reported that glucose deprivation enhanced neuroprotective sirtuin 1 (SIRT1) expression in PC12 cells 21. Therefore, in addition to the negative effects of glucose deprivation, these countervailing systems should also be studied to understand how change in extracellular glucose concentration determines neuron fate.

Reducing environmental glucose levels induces massive changes in intracellular components. Adenosine monophosphate kinase (AMPK) activation is one of the major intracellular responses to glucose deprivation, and can therefore be seen as an intracellular metabolic or energy sensor 22. AMPK is activated during glucose deprivation, mainly by increased cellular consumption of ATPs 23, 24. The activated AMPK then phosphorylates several substrates including cyclic AMP‐responsive element‐binding protein (CREB), thereby activating several transcription factors that facilitate cell adaptation to reduced glucose levels 25. Glucose deprivation also enhances stress‐activated mitogen‐activated protein kinases (MAPKs). For example, Lauretti and Pratico showed that glucose deprivation induced p38 in neuro‐2A (N2A) mouse neuroblastoma cells 26. p38 was also activated in several oxygen/glucose deprivation models 27, 28, 29. Similarly, the other MAPK family member, extracellular signal regulated kinase 1/2 (Erk1/2), was found to be often activated by ischemic‐like insults 29, 30, 31. Nevertheless, further research is required to understand the molecular mechanism or mechanisms underlying this stress‐induced neuroprotection.

The main objective of the present study was to investigate whether and, if so, how glucose deprivation affects *PGRN* expression in PC12 cells, as a model to analyze neuronal stress‐responses and neurosecretions 32, 33, 34. In addition, we also analyzed changes in sortilin expressional, which contributes to reducing extracellular PGRN levels as described above, to better understand the effects of glucose deprivation on integrated PGRN–sortilin systems.

## Materials and methods

### Materials

The western blot detection kit (ECL prime™ detection reagents) was purchased from GE Healthcare Inc. (Rockford, IL, USA); Dulbecco's modified Eagle's medium (DMEM), penicillin/streptomycin, and Trypsin‐EDTA from Nacalai Tesque (Kyoto, Japan); cell culture equipment from Corning Inc. (Corning, NY, USA); and fetal bovine serum (FBS) from BioWest (Nuaillé, France). Unless otherwise noted, all chemicals were of the purest grade available from Nacalai Tesque, Sigma Chemicals (St. Louis, MO, USA) or Wako Pure Chemical Industries, Ltd. (Osaka, Japan). Because only an established cell line (PC12 cell) was used in this study, IRB approval was not required.

### Cell culture

An established rat adrenal pheochromocytoma cell line, PC12 cell, was obtained from Dr. Shin‐Ichiro Takahashi (University of Tokyo, Tokyo, Japan). The PC12 cells were maintained in DMEM (22.5 mm glucose) containing 10% FBS, 30 μg·mL^−1^ penicillin, and 100 μg·mL^−1^ streptomycin at 37 °C under 5% CO_2_ atmosphere. The medium was exchanged every 72 h. For all experiments, cells were grown on six‐well plates (Corning Inc.) at a density of 5 × 10^4^ cells per well in 3 mL growth medium, or on 96‐well plates (Corning Inc.) at a density of 5 × 10^3^ cells per well in 0.2 mL growth medium. Three days after plating, cells typically reached 50–70% confluence (Day 0). Differentiation was then induced by switching to DMEM (22.5 mm glucose) supplemented with 100 ng·mL^−1^ NGF, 30 μg·mL^−1^ penicillin, and 100 μg·mL^−1^ streptomycin.

### Western blotting

The expression and phosphorylation of each protein were analyzed by western blot analysis as previously described 35. Briefly, cells were seeded on six‐well plates at a density of 1 × 10^5^ cells per well, differentiation was induced as described above, and the medium was switched to differentiation medium containing different amounts of glucose or sucrose (0, 5, or 22.5 mm). Cell lysates were prepared using lysis buffer [2% SDS, 1% 2‐mercaptoethanol, 10% glycerol, 0.0033% bromophenol blue, and 50 mm Tris/Cl (pH 6.8)]. These cell lysates were subjected to 12% SDS‐PAGE (1 : 30, bis:acrylamide). Proteins were transferred to a polyvinylidene difluoride membrane (Immobilon‐P; Millipore Corp., Bedford, MA, USA), and the membranes were blocked for 30 min with 3% BSA in Tris‐buffered saline (TBS) containing 0.1% Tween‐20. Each protein was detected with 1‐h incubation with a 1 : 1000 dilution of primary antibodies: anti‐phospho AMPK (Thr172), anti‐AMPK, anti‐phospho Acetyl‐CoA carboxylase (ACC) (Ser79), anti‐ACC, anti‐phospho Erk1/2 (Thr202/Tyr204), anti‐Erk1/2, anti‐phospho p38 (Thr180/Tyr182), anti‐p38, anti‐β‐actin antibodies (Cell Signaling Technology, Danvers, MA, USA), or anti‐sortilin antibodies (Abcam plc., Cambridge, UK). Specific proteins were visualized after subsequent incubation with 1:5000 dilution of anti‐mouse or rabbit IgG conjugated to horseradish peroxidase (Cell Signaling Technology) and an ECL detection procedure (GE Healthcare Inc.). Protein band intensity was quantified using imagej software (National Institutes of Health, Bethesda, MD, USA).

### Quantitative PCR analysis

PC12 cells were differentiated as previously described, and then cultured in DMEM containing different concentrations of glucose (0, 5, or 22.5 mm) for 0–24 h. Total RNA was isolated using a NucleoSpin™ RNA Isolation Kit (Takara Bio Inc., Shiga, Japan) according to the manufacturer's protocol. cDNAs were synthesized from total RNA using ReverTra Ace qPCR RT Master Mix (Toyobo Co. Ltd., Osaka, Japan). Fluorescence real‐time PCR analysis was performed using a Step One instrument (Life Technologies Corporation, Grand Island, NY, USA) and an SYBR Green detection kit according to the manufacture's protocol (KAPA Biosystems Inc., Woburn, MA, USA). PCR primers for measuring each gene comprised: rat *PGRN*, 5′‐CAC TGT CCT GAT GGC TAC TCT TG‐3′ and 5′‐CTA CCA GGA CAC TGG ACA GCA C‐3′; rat *SORT1*, 5′‐GAC ACA TGG AGC ATG GCA CA‐3′ and 5′‐TGC CTC GGT CAT CAG AGG TAA AG‐3′; and rat *GAPDH*, 5′‐GGC ACA GTC AAG GCT GAG AAT G‐3′ and 5′‐ATG GTG GTG AAG ACG CCA GTA‐3′.

### Cell death measurement

PC12 cells were seeded on 96‐well plates and differentiated as previously described 36. Percentage cell death was evaluated using the lactate dehydrogenase Cytotoxicity Detection Kit^PLUS^ (Roche Diagnostics K.K., Basel, Switzerland) according to the manufacturer's protocol.

### Statistical analysis

Comparisons between treatment groups were tested using one‐way ANOVA with Tukey's *post hoc* test or Student's *t*‐test. Differences for which *P* < 0.05 were considered statistically significant.

## Results

### Glucose deprivation induced *PGRN* gene expression

**Figure 1:**
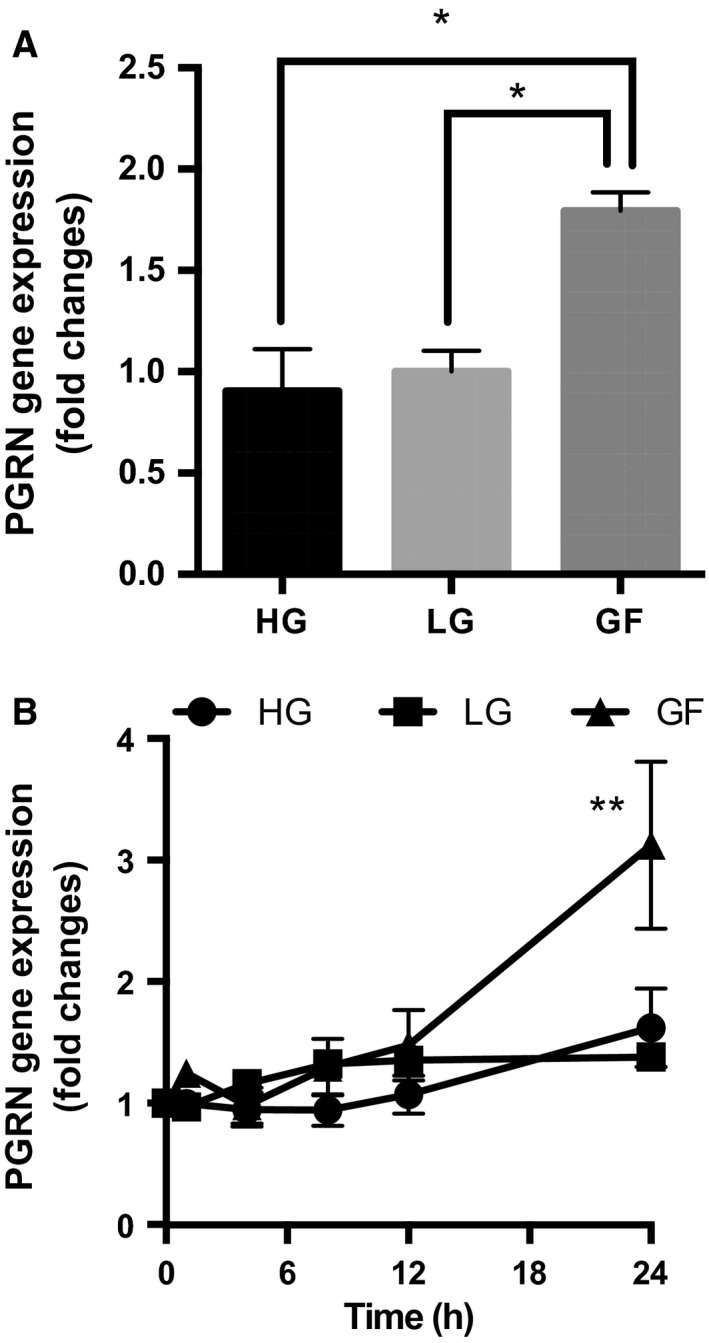
Glucose deprivation induces PGRN gene expression. (A,B) PC12 cells were differentiated in differentiation medium [DMEM (22.5 mm glucose) supplemented with 100 ng·mL^−1^ NGF] for 72 h. (A) The medium was then switched to DMEM [containing different concentrations of glucose: high glucose (HG), 22.5 mm; low glucose (LG), 5 mm; or glucose‐free (GF), 0 mm] supplemented with 100 ng·mL^−1^ NGF and incubated for 24 h. *PGRN* mRNA levels were evaluated by quantitative PCR. Corresponding graph represents fold change in *PGRN* expression normalized to *GAPDH* (LG as control). Data are mean ± SEM (*P < 0.05, *n* = 3, one‐way ANOVA). (B) The cells were cultured under HG, LG, or GF condition for the indicated times, and *PGRN* mRNA levels were measured as described above. Corresponding graph represents fold change in *PGRN* expression normalized to *GAPDH* (time 0 as control). Data are mean ± SEM (**P < 0.01, *n* = 3, one‐way ANOVA).

To determine whether *PGRN* gene expression was modified by changes in glucose availability, differentiated PC12 cells were cultured for 24 h in DMEM containing different concentrations of glucose [high glucose (HG), 22.5 mm; low glucose (LG), 5 mm; or glucose‐free (GF), 0 mm], supplemented with 100 ng·mL^−1^ NGF. *PGRN* gene expression was then monitored by quantitative PCR as described in Materials and methods. As shown in Fig. 1A, *PGRN* gene expression was significantly increased (approximately twofold) when cells were cultured under the GF condition for 24 h (*P < 0.05, *n* = 3) (Fig. 1A). On the other hand, no significant differences in *PGRN* gene expression between the HG and LG groups were detected (Fig. 1A). These results strongly indicate that glucose deprivation (0 mm glucose) contributes to induction of *PGRN* gene, while the glucose‐enriched condition (22.5 mm glucose) did not affect *PGRN* expression compared to the LG condition. Time course experiments revealed that at least 12–24 h of glucose deprivation is necessary for these changes to occur (**P < 0.01, *n* = 3) (Fig. 1B).

### Change in AMPK activation or in osmotic pressure is not involved in glucose deprivation‐induced PGRN gene expression

**Figure 2:**
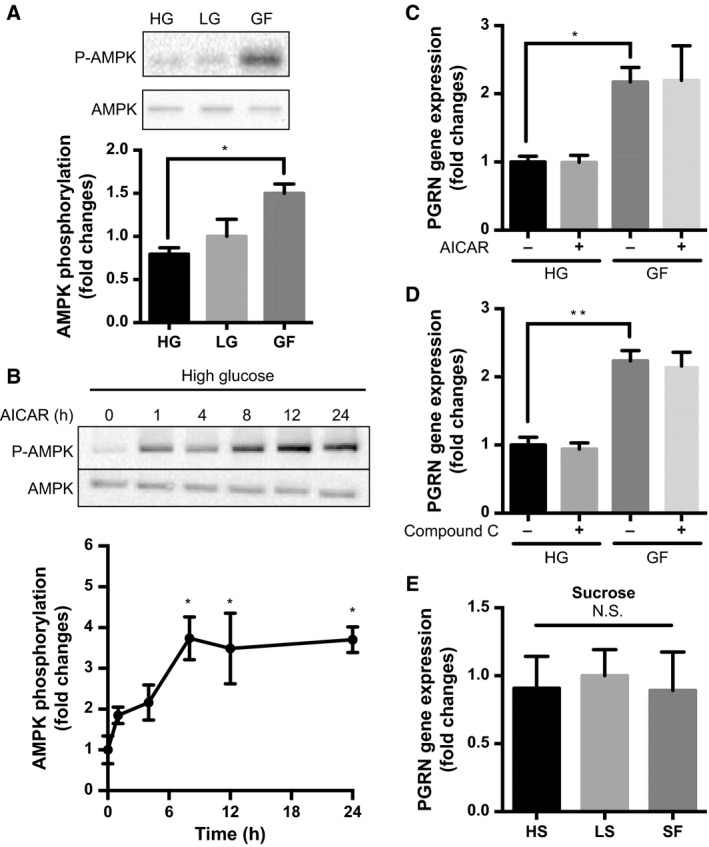
Impact of AMPK activation and osmotic pressure change on PGRN gene expression induced by glucose deprivation. (A) Differentiated PC12 cells were cultured under the HG, LG, or GF conditions for 24 h, and AMPK phosphorylation was evaluated by western blot analysis. Corresponding graph represents fold change in AMPK phosphorylation normalized to total AMPK expression. Data are mean ± SEM (*P < 0.05, *n* = 3, one‐way ANOVA). (B) Differentiated PC12 cells were stimulated with 0.5 mm AICAR under the HG condition for the indicated times, and AMPK phosphorylation was measured as described above. Data are mean ± SEM (*P < 0.05, *n* = 3, one‐way ANOVA). (C) Differentiated PC12 cells were stimulated with 0.5 mm AICAR under the HG or GF condition for 24 h, and *PGRN* gene expression was measured by quantitative PCR (normalized to *GAPDH*). Data are mean ± SEM (*P < 0.05, *n* = 3–5, one‐way ANOVA). (D) Differentiated PC12 cells were cultured under the HG or GF condition in the presence or absence of 0.25 μm compound C for 24 h, and *PGRN* gene expression was measured by quantitative PCR (normalized to *GAPDH*). Data are mean ± SEM (**P < 0.01, *n* = 3–5, one‐way ANOVA). (E) Differentiated PC12 cells were cultured in DMEM + 100 ng·mL^−1^ NGF containing 25, 5, or 0 mm sucrose (HS, LS, or SF, respectively). *PGRN* gene expression was measured by quantitative PCR (normalized to *GAPDH*). Data are mean ± SEM (N.S., nonsignificant; *n* = 4, one‐way ANOVA).

Next, we investigated the mechanisms underlying how glucose deprivation initiates *PGRN* gene expression. As described in the introduction, reduced glucose concentration is known to activate AMPK. Results also confirmed that AMPK phosphorylation, which indicates AMPK activity, was greater under the GF condition compared then under HG (*P < 0.05, *n* = 3) (Fig. 2A). To determine the role of AMPK in *PGRN* gene expression, we tested the effects of AICAR, a potent AMPK activator. Treatment with 0.5 mm AICAR rapidly elevated AMPK phosphorylation under HG condition (*P < 0.05, *n* = 3, compared to 0 h) (Fig. 2B); however, 24 h of AICAR treatment did not elevate the *PGRN* gene expression (*n* = 3–5) (Fig. 2C). We also tested the effects of a specific AMPK inhibitor, compound C, on GF‐induced *PGRN* gene expression; however, the expected effects of compound C were not observed (*n* = 3–5) (Fig. 2D).

As increased glucose concentration in the medium results in increased osmotic pressure, we also tested whether a high concentration of nonmetabolizable sugar (sucrose) affected *PGRN* gene expression in the same way as glucose. Differentiated PC12 cells were cultured for 24 h in DMEM without glucose but with various concentrations of sucrose [high sucrose (HS), 22.5 mm; low sucrose (LS), 5 mm; or sucrose‐free (SF), 0 mm)] supplemented with 100 ng·mL^−1^ NGF, and *PGRN* gene expression was measured. No effect of sucrose on *PGRN* gene expression was observed (*n* = 4) (Fig. 2E), suggesting that change in osmotic pressure plays no significant role, but that metabolic processes and/or intracellular signalings activated by glucose influence change in *PGRN* gene expression.

### p38 MAPK activation contributes to PGRN gene induction by glucose deprivation

**Figure 3:**
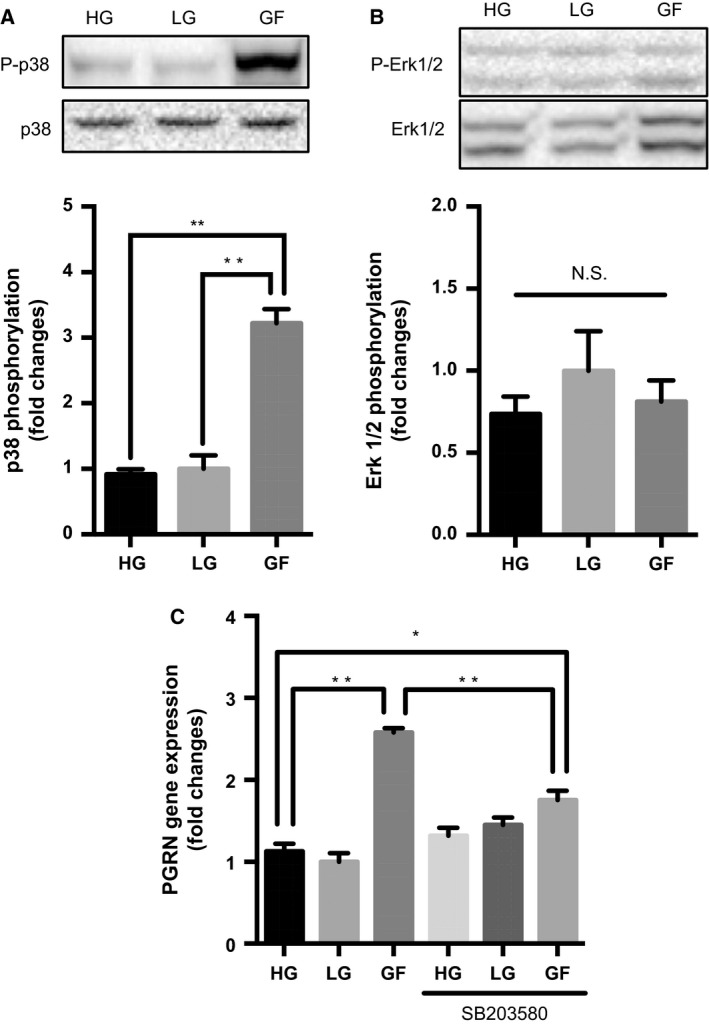
Role of p38 activation in PGRN gene induction by glucose deprivation. (A,B) Differentiated PC12 cells were cultured under the HG, LG, or GF condition for 15 min, and total cell lysates were analyzed by western blot, using anti‐phosphorylated p38 (P‐p38) and total‐p38 antibodies (A), or anti‐phosphorylated Erk1/2 antibodies (P‐Erk1/2) and total‐Erk1/2 antibodies (B). Corresponding graphs represent fold change for each phosphorylation normalized to total expression (LG as control). Data are mean ± SEM (**P < 0.01, *n* = 3, one‐way ANOVA). (C) Differentiated PC12 cells were cultured under the HG, LG, or GF condition in the presence or absence of 5 μm SB203580 for 24 h, and PGRN gene expression was measured by quantitative PCR. Corresponding graph represents fold change in *PGRN* expression normalized to *GAPDH*. Data are mean ± SEM (*P < 0.05, **P < 0.01, *n* = 5, one‐way ANOVA).

We next studied the role of the MAPK family in *PGRN* gene expression, first checking whether the activity of each MAPK could be changed by glucose deprivation. As shown in Fig. 3A, when cells were cultured under the GF condition, p38 activity was significantly elevated [approximately threefold compared to the HG or LG conditions (**P < 0.01, *n* = 3) (Fig. 3A)], whereas no significant change in Erk1/2 phosphorylation was observed according to glucose concentration (*n* = 3) (Fig. 3B).

To evaluate the role of p38 activation by glucose deprivation, differentiated PC12 cells were cultured for 24 h under the HG, LG, or GF conditions in the presence or absence of a p38‐specific inhibitor, SB203580, and then *PGRN* gene expression was measured. As in Fig. 1A, *PGRN* gene expression was significantly elevated under the GF condition, but this effect was abolished in the presence of SB203580 (*P < 0.05, **P < 0.01, *n* = 5) (Fig. 3C), suggesting that glucose deprivation‐induced p38 activation mediates *PGRN* gene induction by glucose deprivation.

### Glucose deprivation also alters expression of the PGRN receptor, sortilin

**Figure 4:**
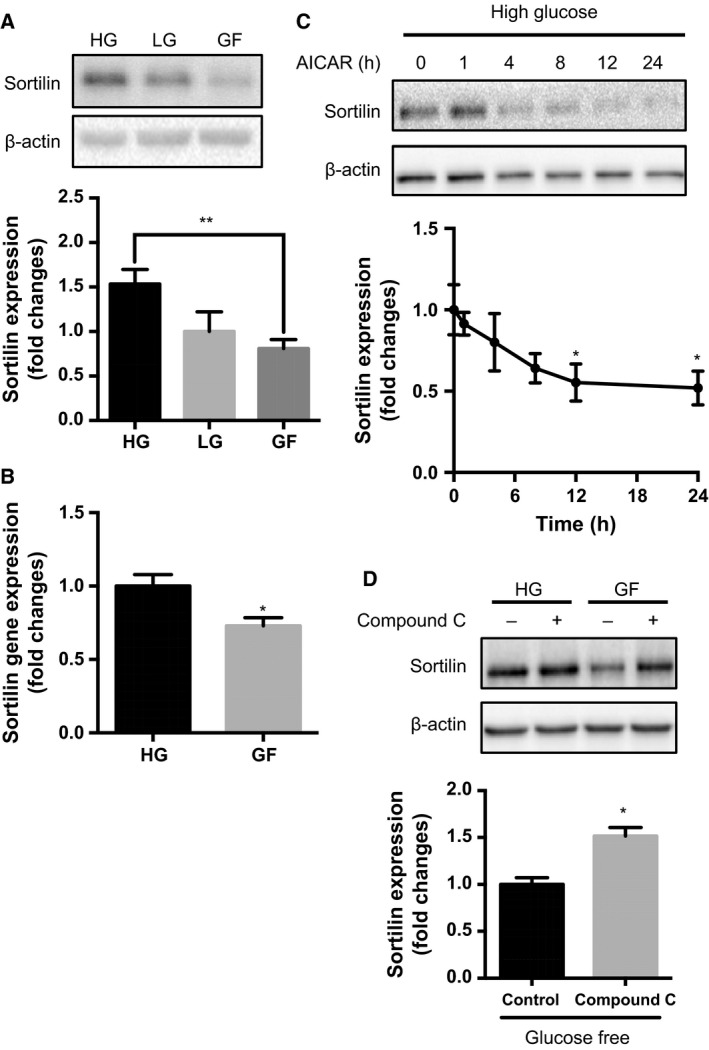
Glucose deprivation decreases sortilin via AMPK activation. (A,B) Differentiated PC12 cells were cultured under the HG, LG, or GF condition for 24 h. (A) Sortilin protein levels were measured by western blot. Corresponding graph represents fold change in sortilin expression normalized to β‐actin. Data are mean ± SEM (**P < 0.01, *n* = 7, one‐way ANOVA). (B) *SORT1* expression was measured by quantitative PCR normalized to *GAPDH*. Data are mean ± SEM (*P < 0.05, *n* = 3, *t*‐test). (C) Differentiated PC12 cells were stimulated with 0.5 mm AICAR under the HG condition for the indicated times, and sortilin expression was measured by western blot. Corresponding graph represents fold change in sortilin expression normalized to β‐actin. Data are mean ± SEM (*P < 0.05, *n* = 3, one‐way ANOVA). (D) Differentiated PC12 cells were cultured under the GF condition in the presence or absence of 0.25 μm compound C for 24 h, and sortilin expression was measured by western blot. Corresponding graph represents fold change in sortilin expression normalized to β‐actin. Data are mean ± SEM (*P < 0.05, *n* = 3, *t*‐test).

We also examined whether changing glucose concentration in the medium would modify the expression of sortilin, identified as a PGRN receptor and implicated in PGRN endocytosis. Differentiated PC12 cells were again cultured for 24 h in medium containing different amounts of glucose, and the cell lysate underwent western blot analysis using anti‐sortilin antibody. Sortilin protein levels were significantly decreased (approximately 0.5‐fold) when cells were exposed to the GF condition compared to the HG group (**P < 0.01, *n* = 7) (Fig. 4A). In addition, sortilin gene (*SORT1*) expression was also decreased under the GF condition, suggesting that the decrease in sortilin protein level was at least partly controlled by mRNA level (*P < 0.05, *n* = 3) (Fig. 4B). Interestingly, the decrease in sortilin protein level under the GF condition seemed to be regulated by AMPK, as pharmacological activation of AMPK by AICAR under the HG condition significantly decreased sortilin levels in a time‐dependent manner (*P < 0.05, *n* = 3) (Fig. 4C). The data also confirmed that inhibition of AMPK by compound C under the GF condition increased sortilin levels (*P < 0.05, *n* = 3) (Fig. 4D).

## Discussion

### Glucose deprivation increases PGRN gene expression via p38 activation

Detrimental environments, such as glucose deprivation, are stressors for cells; at the same time, such environmental conditions activate several cell protection mechanisms. The present study demonstrated that reduction of glucose supplements induced *PGRN* gene expression. Moreover, expression of sortilin, implicated in PGRN endocytosis, was decreased by glucose deprivation, which potentially increases extracellular PGRN concentration. Overall, it could be hypothesized that glucose deprivation increased PGRN levels via two distinct mechanisms.

As described above, numerous reports have shown that PGRN has a neuroprotective property 4, 6, 15, and the present study confirmed that PGRN pretreatment attenuates PC12 cell death induced by high hydrogen peroxide concentrations (Fig. S1). Furthermore, recent reports strongly suggested that PGRN was a stress‐responsive factor in many cell types. All of these findings support the present hypothesis that different types of stressor induce neuroprotective PGRN expression. Although further investigations are required to know whether all stressors stimulate *PGRN* gene expression via the same mechanism or mechanisms, the present study showed that p38 activation induced by glucose deprivation plays central role in this phenomenon.

It has been reported that glucose deprivation induces p38 phosphorylation in many cell types. The present study also clearly demonstrated that glucose deprivation stimulated p38 activation in differentiated PC12 cells (Fig. 3A). On the other hand, some reports showed that p38 was activated by high concentrations of glucose, for instance in SHSY5Y human neuroblastoma cells 37. Thus, the regulatory system of p38 activation by glucose may be dependent on cell type. It is noteworthy that p38 is activated not only by glucose deprivation but also by various stressors, such as hypoxia, ultraviolet irradiation, heat shock, etc. 38, 39, 40, 41, which are known to stimulate PGRN expression, as mentioned above. The present study is the first to report that stress‐dependent p38 activation contributes to *PGRN* gene expression; however, intriguingly, Wang *et al*. recently reported that gastric cancer cells coming in contact with live *Helicobacter pylori* induced *PGRN* expression associated with p38 activation 42.

The next important question is to identify the downstream target or targets of p38 that underlie PGRN expression. There are several signal‐transducing p38 substrates, such as MAP kinase‐activated protein kinases (M2 and M3), heat shock protein 27 (HSP27), CREB, activating transcription factors, and more 43, 44, 45. A chemical compound termed ‘crebinostat’ that robustly activates CREB‐mediated transcription was shown to substantially up‐regulate *PGRN* gene expression 46. Further studies may reveal that p38‐dependent CREB phosphorylation also plays a critical role in glucose deprivation‐dependent *PGRN* gene induction.

As described, we identified glucose deprivation‐dependent p38 activation as a major signal regulating *PGRN* gene induction (Fig. 3C); however, the significant increases in *PGRN* gene expression induced by glucose deprivation were still observed even in the presence of 5 μm SB203580, a concentration which was sufficient to inhibit p38 activity (data not shown) (Fig. 3C). These results suggested that the other signaling molecule(s) might also participate in the glucose‐dependent *PGRN* expression changes.

### Glucose deprivation decreases sortilin levels via AMPK activation

The present study found that expression of sortilin, one of the PGRN receptors, was significantly reduced by glucose deprivation. We recently discovered similar glucose‐dependent control of sortilin expression in skeletal muscle cells 47. Moreover, sortilin expression in liver was repressed in both genetic (ob/ob) mice and high‐fat diet models 48, and control of sortilin expression may thus vary between tissues and organs. As described above, sortilin was reported to be a key receptor for PGRN, and induced endocytosis and lysosomal transfer of PGRN 10. In this report, the authors expressed sortilin in COS‐7 cells and applied fluorescent‐labeled PGRN to examine their kinetics by using several cell biological techniques. They clearly showed there was rapid endocytosis of extracellular PGRN by cell surface sortilin to COS‐7 lysosomes. Moreover, they also showed that brain and serum PGRN levels were significantly increased in mice lacking sortilin 10. Similarly, a genome‐wide screen suggested that sortilin expression regulated PGRN levels in human plasma 49. Overall, extracellular levels of PGRN seem to be at least partly determined by sortilin‐mediated endocytosis. The present study attempted to measure PGRN levels in cellular and conditioned medium obtained from PC12 cell culture, but levels were not detectable either by ELISA or western blot analysis (data not shown). On the other hand, it is possible that PGRN concentration in the pericellular region of the cells can be changed by glucose deprivation, which is difficult to evaluate by these classical methods. We are therefore conducting further research to detect pericellular PGRN concentration by other methods.

Intriguingly, the function of sortilin is not limited to PGRN action. Sortilin makes a complex with p75 neurotrophin receptor (p75NTR), which triggers proNGF‐ or proBDNF‐induced neuronal apoptosis 50, 51. Hence, this glucose‐dependent sortilin regulation may modify not only PGRN function but also the biological function of neurotrophin precursors. Furthermore, sortilin‐deficient mice showed reduced neuronal apoptosis 52, and it has also been reported that expression of proNGF and sortilin was increased in aging rodent basal forebrain and sympathetic neurons 53.

Despite these important roles of sortilin in the CNS, the detailed molecular mechanisms of sortilin expression remain elusive. Saadipour *et al*. recently demonstrated that amyloid‐β_42_ treatment of SH‐SY5Y human neuroblastoma cells significantly induced sortilin expression, which is mediated by Rho‐associated protein kinase (ROCK) 54. The present study showed significant AMPK activation and sortilin reduction in the GF compared to the HG condition (Fig. 4A). In addition to this correlation, the present pharmacological experiments also suggested an involvement of AMPK activity in sortilin expression (Fig. 4C,D). Coincidently, several reports indicated that AMPK activation has negative effects on RhoA‐ROCK signaling in vascular smooth‐muscle cells 55, 56. Further studies may reveal the precise molecular mechanism(s) of neuronal sortilin expression, with potentially strong impact on neuronal fate.

## Conclusion

Because PGRN haploinsufficiency is a crucial factor in some types of neurodegenerative disease, studies of the expressional control of PGRN are extremely important. Although further investigation is required, the present study suggested that decreased glucose levels around PC12 cells can modify PGRN properties via two distinct mechanisms: p38‐dependent induction of *PGRN* expression, and reduction of sortilin levels, controlling AMPK‐dependent PGRN endocytosis. Taken together with the fact that PGRN has a neuroprotective action on PC12 cells, this integrated system may shield cell survival from detrimental stress such as glucose deprivation.

## Author contributions

KIK and TN mainly constructed the concept for this research and designed the experiments. HK, NM, JL, and MN helped design and implement the study. KIK, YI, MK, SK, and HS collected, processed, and analyzed data, and also contributed to the literature search. TN wrote the manuscript, with critical revision for important intellectual content from HK, NM, JL, and MN.

## Supporting information


**Fig. S1.** PGRN pretreatment attenuates H_2_O_2_‐induced PC12 cell death.

## References

[1] Cruts M , Gijselinck I , van der Zee J , Engelborghs S , Wils H , Pirici D , Rademakers R , Vandenberghe R , Dermaut B , Martin JJ *et al* (2006) Null mutations in progranulin cause ubiquitin‐positive frontotemporal dementia linked to chromosome 17q21. Nature 442, 920–924. 16862115 10.1038/nature05017

[2] Baker M , Mackenzie IR , Pickering‐Brown SM , Gass J , Rademakers R , Lindholm C , Snowden J , Adamson J , Sadovnick AD , Rollinson S *et al* (2006) Mutations in progranulin cause tau‐negative frontotemporal dementia linked to chromosome 17. Nature 442, 916–919. 16862116 10.1038/nature05016

[3] Irwin D , Lippa CF and Rosso A (2009) Progranulin (PGRN) expression in ALS: an immunohistochemical study. J Neurol Sci 276, 9–13. 18848708 10.1016/j.jns.2008.08.024

[4] Van Damme P , Van Hoecke A , Lambrechts D , Vanacker P , Bogaert E , van Swieten J , Carmeliet P , Van Den Bosch L and Robberecht W (2008) Progranulin functions as a neurotrophic factor to regulate neurite outgrowth and enhance neuronal survival. J Cell Biol 181, 37–41. 18378771 10.1083/jcb.200712039 PMC2287280

[5] Ryan CL , Baranowski DC , Chitramuthu BP , Malik S , Li Z , Cao M , Minotti S , Durham HD , Kay DG , Shaw CA *et al* (2009) Progranulin is expressed within motor neurons and promotes neuronal cell survival. BMC Neurosci 10, 130. 19860916 10.1186/1471-2202-10-130 PMC2779192

[6] Xu J , Xilouri M , Bruban J , Shioi J , Shao Z , Papazoglou I , Vekrellis K and Robakis NK (2011) Extracellular progranulin protects cortical neurons from toxic insults by activating survival signaling. Neurobiol Aging 32, 2326.e5–2326.e16. 10.1016/j.neurobiolaging.2011.06.017 PMC3375317 21820214

[7] Filiano AJ , Martens LH , Young AH , Warmus BA , Zhou P , Diaz‐Ramirez G , Jiao J , Zhang Z , Huang EJ , Gao FB *et al* (2013) Dissociation of frontotemporal dementia‐related deficits and neuroinflammation in progranulin haploinsufficient mice. J Neurosci 33, 5352–5361. 23516300 10.1523/JNEUROSCI.6103-11.2013 PMC3740510

[8] Martens LH , Zhang J , Barmada SJ , Zhou P , Kamiya S , Sun B , Min SW , Gan L , Finkbeiner S , Huang EJ *et al* (2012) Progranulin deficiency promotes neuroinflammation and neuron loss following toxin‐induced injury. J Clin Invest 122, 3955–3959. 23041626 10.1172/JCI63113 PMC3484443

[9] Tanaka Y , Matsuwaki T , Yamanouchi K and Nishihara M (2013) Exacerbated inflammatory responses related to activated microglia after traumatic brain injury in progranulin‐deficient mice. Neuroscience 231, 49–60. 23201826 10.1016/j.neuroscience.2012.11.032

[10] Hu F , Padukkavidana T , Vægter CB , Brady OA , Zheng Y , Mackenzie IR , Feldman HH , Nykjaer A and Strittmatter SM (2010) Sortilin‐mediated endocytosis determines levels of the frontotemporal dementia protein, progranulin. Neuron 68, 654–667. 21092856 10.1016/j.neuron.2010.09.034 PMC2990962

[11] Gass J , Lee WC , Cook C , Finch N , Stetler C , Jansen‐West K , Lewis J , Link CD , Rademakers R , Nykjær A *et al* (2012) Progranulin regulates neuronal outgrowth independent of sortilin. Proc Natl Acad Sci USA 109, 21510–21515. 22781549 10.1186/1750-1326-7-33 PMC3508877

[12] De Muynck L , Herdewyn S , Beel S , Scheveneels W , Van Den Bosch L , Robberecht W and Van Damme P (2013) The neurotrophic properties of progranulin depend on the granulin E domain but do not require sortilin binding. Neurobiol Aging 34, 2541–2547. 23706646 10.1016/j.neurobiolaging.2013.04.022

[13] Guerra RR , Kriazhev L , Hernandez‐Blazquez FJ and Bateman A (2007) Progranulin is a stress‐response factor in fibroblasts subjected to hypoxia and acidosis. Growth Factors 25, 280–285. 18092235 10.1080/08977190701781222

[14] Piscopo P , Rivabene R , Adduci A , Mallozzi C , Malvezzi‐Campeggi L , Crestini A and Confaloni A (2010) Hypoxia induces up‐regulation of progranulin in neuroblastoma cell lines. Neurochem Int 57, 893–898. 20933034 10.1016/j.neuint.2010.09.008

[15] Sato K , Yamanaka Y , Ishii M , Ishibashi K , Ogura Y , Ohtani‐Kaneko R , Nishihara M and Nedachi T (2014) Dual cell protective mechanisms activated by differing levels of oxidative stress in HT22 murine hippocampal cells. Biosci Biotechnol Biochem 78, 1495–1503. 25060136 10.1080/09168451.2014.936343

[16] Suzuki A , Kusakai G , Kishimoto A , Lu J , Ogura T and Esumi H (2003) ARK5 suppresses the cell death induced by nutrient starvation and death receptors via inhibition of caspase 8 activation, but not by chemotherapeutic agents or UV irradiation. Oncogene 22, 6177–6182. 13679856 10.1038/sj.onc.1206899

[17] Vander Heiden MG , Plas DR , Rathmell JC , Fox CJ , Harris MH and Thompson CB (2001) Growth factors can influence cell growth and survival through effects on glucose metabolism. Mol Cell Biol 21, 5899–5912. 11486029 10.1128/MCB.21.17.5899-5912.2001 PMC87309

[18] Gonin‐Giraud S , Mathieu AL , Diocou S , Tomkowiak M , Delorme G and Marvel J (2002) Decreased glycolytic metabolism contributes to but is not the inducer of apoptosis following IL‐3‐starvation. Cell Death Differ 9, 1147–1157. 12232803 10.1038/sj.cdd.4401079

[19] Lee YJ , Galoforo SS , Berns CM , Tong WP , Kim HR and Corry PM (1997) Glucose deprivation‐induced cytotoxicity in drug resistant human breast carcinoma MCF‐7/ADR cells: role of c‐myc and bcl‐2 in apoptotic cell death. J Cell Sci 110 (Pt 5), 681–686. 9092950 10.1242/jcs.110.5.681

[20] Ebel D , Redler S , Preckel B , Schlack W and Thämer V (2005) Moderate glucose deprivation preconditions myocardium against infarction. Horm Metab Res 37, 516–520. 16138266 10.1055/s-2005-870321

[21] Fujino K , Ogura Y , Sato K and Nedachi T (2013) Potential neuroprotective effects of SIRT1 induced by glucose deprivation in PC12 cells. Neurosci Lett 557 (Pt B): 148–153 24183892 10.1016/j.neulet.2013.10.050

[22] Shirwany NA and Zou MH (2014) AMPK: a cellular metabolic and redox sensor. A minireview. Front Biosci (Landmark Ed) 19, 447–474. 24389195 10.2741/4218 PMC4101001

[23] Hutber CA , Hardie DG and Winder WW (1997) Electrical stimulation inactivates muscle acetyl‐CoA carboxylase and increases AMP‐activated protein kinase. Am J Physiol 272 (Pt 1), E262–E266. 9124333 10.1152/ajpendo.1997.272.2.E262

[24] Vavvas D , Apazidis A , Saha AK , Gamble J , Patel A , Kemp BE , Witters LA and Ruderman NB (1997) Contraction‐induced changes in acetyl‐CoA carboxylase and 5′‐AMP‐activated kinase in skeletal muscle. J Biol Chem 272, 13255–13261. 9148944 10.1074/jbc.272.20.13255

[25] Towler MC and Hardie DG (2007) AMP‐activated protein kinase in metabolic control and insulin signaling. Circ Res 100, 328–341. 17307971 10.1161/01.RES.0000256090.42690.05

[26] Lauretti E and Praticò D (2015) Glucose deprivation increases tau phosphorylation via P38 mitogen‐activated protein kinase. Aging Cell 14, 1067–1074. 26219917 10.1111/acel.12381 PMC4693472

[27] Tabakman R , Jiang H , Schaefer E , Levine RA and Lazarovici P (2004) Nerve growth factor pretreatment attenuates oxygen and glucose deprivation‐induced c‐Jun amino‐terminal kinase 1 and stress‐activated kinases p38alpha and p38beta activation and confers neuroprotection in the pheochromocytoma PC12 Model. J Mol Neurosci 22, 237–250. 14997018 10.1385/jmn:22:3:237

[28] Shinozaki Y , Sato Y , Koizumi S , Ohno Y , Nagao T and Inoue K (2007) Retinoic acids acting through retinoid receptors protect hippocampal neurons from oxygen‐glucose deprivation‐mediated cell death by inhibition of c‐jun‐N‐terminal kinase and p38 mitogen‐activated protein kinase. Neuroscience 147, 153–163. 17521827 10.1016/j.neuroscience.2007.04.032

[29] Strassburger M , Braun H and Reymann KG (2008) Anti‐inflammatory treatment with the p38 mitogen‐activated protein kinase inhibitor SB239063 is neuroprotective, decreases the number of activated microglia and facilitates neurogenesis in oxygen‐glucose‐deprived hippocampal slice cultures. Eur J Pharmacol 592, 55–61. 18638472 10.1016/j.ejphar.2008.06.099

[30] Tabakman R , Jiang H , Shahar I , Arien‐Zakay H , Levine RA and Lazarovici P (2005) Neuroprotection by NGF in the PC12 in vitro OGD model: involvement of mitogen‐activated protein kinases and gene expression. Ann N Y Acad Sci 1053, 84–96. 16179511 10.1196/annals.1344.008

[31] Zhang D and Wood CE (2005) Neuronal prostaglandin endoperoxide synthase 2 responses to oxygen and glucose deprivation are mediated by mitogen‐activated protein kinase ERK1/2. Brain Res 1060, 100–107. 16185670 10.1016/j.brainres.2005.08.033

[32] Tabakman R , Lazarovici P and Kohen R (2002) Neuroprotective effects of carnosine and homocarnosine on pheochromocytoma PC12 cells exposed to ischemia. J Neurosci Res 68, 463–469. 11992473 10.1002/jnr.10228

[33] Li G , Ma R , Huang C , Tang Q , Fu Q , Liu H , Hu B and Xiang J (2008) Protective effect of erythropoietin on beta‐amyloid‐induced PC12 cell death through antioxidant mechanisms. Neurosci Lett 442, 143–147. 18634846 10.1016/j.neulet.2008.07.007

[34] Westerink RH and Ewing AG (2008) The PC12 cell as model for neurosecretion. Acta Physiol (Oxf) 192, 273–285. 18005394 10.1111/j.1748-1716.2007.01805.x PMC2663028

[35] Sato K , Yamanaka Y , Asakura Y and Nedachi T (2016) Glutamate levels control HT22 murine hippocampal cell death by regulating biphasic patterns of Erk1/2 activation: role of metabolic glutamate receptor 5. Biosci Biotechnol Biochem 80, 712–718. 26539627 10.1080/09168451.2015.1107466

[36] Ogura Y , Sato K , Kawashima K , Kobayashi N , Imura S , Fujino K , Kawaguchi H and Nedachi T (2014) Subtoxic levels of hydrogen peroxide induce brain‐derived neurotrophic factor expression to protect PC12 cells. BMC Res Notes 7, 840. 25424467 10.1186/1756-0500-7-840 PMC4256810

[37] Cao M , Jiang J , Du Y and Yan P (2012) Mitochondria‐targeted antioxidant attenuates high glucose‐induced P38 MAPK pathway activation in human neuroblastoma cells. Mol Med Rep 5, 929–934. 22245807 10.3892/mmr.2012.746 PMC3493100

[38] Kim M , Li YX , Dewapriya P , Ryu B and Kim SK (2013) Floridoside suppresses pro‐inflammatory responses by blocking MAPK signaling in activated microglia. BMB Rep 46, 398–403. 23977987 10.5483/BMBRep.2013.46.8.237 PMC4133907

[39] Schieven GL (2005) The biology of p38 kinase: a central role in inflammation. Curr Top Med Chem 5, 921–928. 16178737 10.2174/1568026054985902

[40] Rajashekhar G , Kamocka M , Marin A , Suckow MA , Wolter WR , Badve S , Sanjeevaiah AR , Pumiglia K , Rosen E and Clauss M (2011) Pro‐inflammatory angiogenesis is mediated by p38 MAP kinase. J Cell Physiol 226, 800–808. 20803566 10.1002/jcp.22404

[41] Clark AR and Dean JL (2012) The p38 MAPK pathway in rheumatoid arthritis: a sideways look. Open Rheumatol J 6, 209–219. 23028406 10.2174/1874312901206010209 PMC3460412

[42] Wang H , Sun Y , Liu S , Yu H , Li W , Zeng J , Chen C and Jia J (2011) Upregulation of progranulin by *Helicobacter pylori* in human gastric epithelial cells via p38MAPK and MEK1/2 signaling pathway: role in epithelial cell proliferation and migration. FEMS Immunol Med Microbiol 63, 82–92. 21707777 10.1111/j.1574-695X.2011.00833.x

[43] Yang Y , Kim SC , Yu T , Yi YS , Rhee MH , Sung GH , Yoo BC and Cho JY (2014) Functional roles of p38 mitogen‐activated protein kinase in macrophage‐mediated inflammatory responses. Mediators Inflamm 2014, 352371. 24771982 10.1155/2014/352371 PMC3977509

[44] Guay J , Lambert H , Gingras‐Breton G , Lavoie JN , Huot J and Landry J (1997) Regulation of actin filament dynamics by p38 map kinase‐mediated phosphorylation of heat shock protein 27. J Cell Sci 110 (Pt 3), 357–368. 9057088 10.1242/jcs.110.3.357

[45] Iordanov M , Bender K , Ade T , Schmid W , Sachsenmaier C , Engel K , Gaestel M , Rahmsdorf HJ and Herrlich P (1997) CREB is activated by UVC through a p38/HOG‐1‐dependent protein kinase. EMBO J 16, 1009–1022. 9118940 10.1093/emboj/16.5.1009 PMC1169701

[46] Fass DM , Reis SA , Ghosh B , Hennig KM , Joseph NF , Zhao WN , Nieland TJ , Guan JS , Kuhnle CE , Tang W *et al* (2013) Crebinostat: a novel cognitive enhancer that inhibits histone deacetylase activity and modulates chromatin‐mediated neuroplasticity. Neuropharmacology 64, 81–96. 22771460 10.1016/j.neuropharm.2012.06.043 PMC3447535

[47] Ariga M , Yoneyama Y , Fukushima T , Ishiuchi Y , Ishii T , Sato H , Hakuno F , Nedachi T and Takahashi SI (in press) Glucose deprivation attenuates sortilin levels in skeletal muscle cells. Endocr J. 10.1507/endocrj.EJ16-0319 27980238

[48] Ai D , Baez JM , Jiang H , Conlon DM , Hernandez‐Ono A , Frank‐Kamenetsky M , Milstein S , Fitzgerald K , Murphy AJ , Woo CW *et al* (2012) Activation of ER stress and mTORC1 suppresses hepatic sortilin‐1 levels in obese mice. J Clin Invest 122, 1677–1687. 22466652 10.1172/JCI61248 PMC3336989

[49] Carrasquillo MM , Nicholson AM , Finch N , Gibbs JR , Baker M , Rutherford NJ , Hunter TA , DeJesus‐Hernandez M , Bisceglio GD , Mackenzie IR *et al* (2010) Genome‐wide screen identifies rs646776 near sortilin as a regulator of progranulin levels in human plasma. Am J Hum Genet 87, 890–897. 21087763 10.1016/j.ajhg.2010.11.002 PMC2997361

[50] Nykjaer A , Lee R , Teng KK , Jansen P , Madsen P , Nielsen MS , Jacobsen C , Kliemannel M , Schwarz E , Willnow TE *et al* (2004) Sortilin is essential for proNGF‐induced neuronal cell death. Nature 427, 843–848. 14985763 10.1038/nature02319

[51] Teng HK , Teng KK , Lee R , Wright S , Tevar S , Almeida RD , Kermani P , Torkin R , Chen ZY , Lee FS *et al* (2005) ProBDNF induces neuronal apoptosis via activation of a receptor complex of p75NTR and sortilin. J Neurosci 25, 5455–5463. 15930396 10.1523/JNEUROSCI.5123-04.2005 PMC6724992

[52] Jansen P , Giehl K , Nyengaard JR , Teng K , Lioubinski O , Sjoegaard SS , Breiderhoff T , Gotthardt M , Lin F , Eilers A *et al* (2007) Roles for the pro‐neurotrophin receptor sortilin in neuronal development, aging and brain injury. Nat Neurosci 10, 1449–1457. 17934455 10.1038/nn2000

[53] Al‐Shawi R , Hafner A , Olsen J , Chun S , Raza S , Thrasivoulou C , Lovestone S , Killick R , Simons P and Cowen T (2008) Neurotoxic and neurotrophic roles of proNGF and the receptor sortilin in the adult and ageing nervous system. Eur J Neurosci 27, 2103–2114. 18412630 10.1111/j.1460-9568.2008.06152.x

[54] Saadipour K , Yang M , Lim Y , Georgiou K , Sun Y , Keating D , Liu J , Wang YR , Gai WP , Zhong JH *et al* (2013) Amyloid beta_1‐42_ (Aβ_42_) up‐regulates the expression of sortilin via the p75(NTR)/RhoA signaling pathway. J Neurochem 127, 152–162. 23895422 10.1111/jnc.12383

[55] Gayard M , Guilluy C , Rousselle A , Viollet B , Henrion D , Pacaud P , Loirand G and Rolli‐Derkinderen M (2011) AMPK alpha 1‐induced RhoA phosphorylation mediates vasoprotective effect of estradiol. Arterioscler Thromb Vasc Biol 31, 2634–2642. 21852563 10.1161/ATVBAHA.111.228304

[56] Cao X , Luo T , Luo X and Tang Z (2014) Resveratrol prevents AngII‐induced hypertension via AMPK activation and RhoA/ROCK suppression in mice. Hypertens Res 37, 803–810. 24965170 10.1038/hr.2014.90

