# Supplementary material for: Glucose deprivation regulates the progranulin–sortilin axis in PC12 cells
Source: FEBS Open Bio. 2016 Dec 22;7(2):149–59. doi: 10.1002/2211-5463.12164 (PMC5292667; doi:10.1002/2211-5463.12164)
Supplement: Supplementary file 1 — Fig. S1. PGRN pretreatment attenuates H2O2‐induced PC12 cell death. [file FEB4-7-149-s001.pdf]

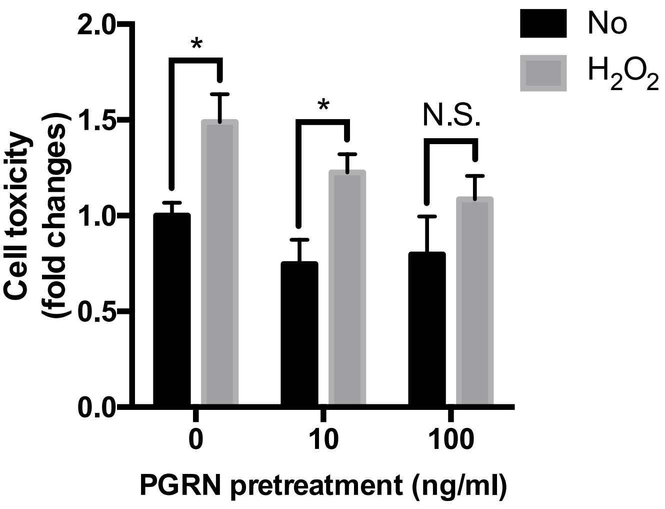

Supplementary Figure 1. PGRN pretreatment attenuates H_2O_2 induced PC12 cell death. (A) Differentiated PC12 cells were pretreated with the indicated amounts of PGRN for 24 h. After washing cells three times with PBS(-), the cells are subsequently treated with 0 uM or 250 uM H_2O_2 for 15 hours. Cell toxicity was measured by LDH assay as described. Data shown represent mean ± SEM, tested using t-test (*$p < 0.05$, $n = 4$) (N.S.; no significant differences are observed).
